# A New Data Analysis System to Quantify Associations between Biochemical Parameters of Chronic Kidney Disease-Mineral Bone Disease

**DOI:** 10.1371/journal.pone.0146801

**Published:** 2016-01-25

**Authors:** Mariano Rodriguez, M. Dolores Salmeron, Alejandro Martin-Malo, Carlo Barbieri, Flavio Mari, Rafael I. Molina, Pedro Costa, Pedro Aljama

**Affiliations:** 1 Nephrology Service, Hospital Reina Sofia, IMIBIC, University of Cordoba, Cordoba, Spain; 2 Fresenius Medical Care, Bad Homburg, Germany; 3 Fresenius Medical Care, Madrid, Spain; University Medical Center Groningen and University of Groningen, NETHERLANDS

## Abstract

**Background:**

In hemodialysis patients, deviations from KDIGO recommended values of individual parameters, phosphate, calcium or parathyroid hormone (PTH), are associated with increased mortality. However, it is widely accepted that these parameters are not regulated independently of each other and that therapy aimed to correct one parameter often modifies the others. The aim of the present study is to quantify the degree of association between parameters of chronic kidney disease and mineral bone disease (CKD-MBD).

**Methods:**

Data was extracted from a cohort of 1758 adult HD patients between January 2000 and June 2013 obtaining a total of 46.141 records (10 year follow-up). We used an advanced data analysis system called Random Forest (RF) which is based on self-learning procedure with similar axioms to those utilized for the development of artificial intelligence. This new approach is particularly useful when the variables analyzed are closely dependent to each other.

**Results:**

The analysis revealed a strong association between PTH and phosphate that was superior to that of PTH and Calcium. The classical linear regression analysis between PTH and phosphate shows a correlation coefficient is 0.27, p<0.001, the possibility to predict PTH changes from phosphate modification is marginal. Alternatively, RF assumes that changes in phosphate will cause modifications in other associated variables (calcium and others) that may also affect PTH values. Using RF the correlation coefficient between changes in serum PTH and phosphate is 0.77, p<0.001; thus, the power of prediction is markedly increased. The effect of therapy on biochemical variables was also analyzed using this RF.

**Conclusion:**

Our results suggest that the analysis of the complex interactions between mineral metabolism parameters in CKD-MBD may demand a more advanced data analysis system such as RF.

## Introduction

In hemodialysis (HD) patients, increased mortality is in part explained by the presence of Chronic Kidney Disease-Mineral and Bone Disorder (CKD-MBD). Deviations of serum concentration of phosphate (P), calcium (Ca) or parathyroid hormone (PTH) from the values recommended by KDIGO (Kidney Disease Improving Global Outcomes) [1,2] are associated with a negative outcome [3–7]. There are a number therapeutic strategies aimed to correct the concentration of these parameters; certainly the rate of success in controlling these parameters is variable [8–12].

The regulation of these three parameters, Ca, P and PTH are not independent from each other [9]. A strategy designed to change and correct the value of one of these parameters may be associated with a divergent effect in one or two of the remaining parameters. Based on our understanding on the regulation of mineral metabolism, one could predict that the modification of a single parameter should be followed by a change in another parameter, which in turn is conditioned by the third parameter. Furthermore, the interrelationship among these three parameters is likely to be non-linear. In a given patient, excessive administration of calcium based phosphate binders may reduce serum P level but it may also increase serum Ca and reduce PTH [13] Thus final result is that in a population of hemodialysis (HD) patients the concentration of these three parameters is the result of both an abnormal mineral metabolism and the treatment used to correct these parameters.

Classical statistical methods may not be optimal for the analysis of non-linear associations among variables simultaneously affected by non-trivial feedback loops. Non-trivial feedback loops refers to a situation in which one variable (such as PTH) causes a variation in a second variable (i.e phosphate) which in turn causes a variation on a third variable (i.e calcium) that can modifies the first variable and so on. And, these variations are not linear. In such a case the use of machine learning techniques can overcome these difficulties [14]. If a sufficiently large amount of data is provided, machine learning techniques (like Random Forest) are capable to generate robust mathematical models that codify relationships among variables [14, 15]. The reliability of these relationships is based on the fact that these relationships emerge directly from the data presented with no *a priori* assumptions [14].

The availability of large datasets is critical for machine learning techniques to work properly [16,17]. Therefore, in medical field, the interest in machine learning approaches is progressively growing due to the availability of electronic health records [15]. In the present study, the data analysis has been performed using a machine learning algorithm called Random Forest (RF) [16] toward a predictive analytic approach. To our knowledge this approach in CKD-MBD context is highly innovative. Nevertheless a few studies have been published in the field of nephrology showing the usefulness of RF to predict the risk of diabetic kidney disease [18], to identify biomarkers that predict kidney transplant outcome [19] and to analyze mRNAs in urine samples of kidney transplant recipients [20].

The objective of the present work was to analyze the complex interrelationships between serum concentrations of Ca, P and PTH in HD patients using the machine learning technique RF for data analysis. This study was not designed to investigate new mechanisms and factors involved in CKD-MBD.

## Methods

### Dataset Description

Data was extracted from a cohort of 1758 adult HD patients between January 1, 2000 and June 1, 2013 in the area of Cordoba. Patients belonged to 7 hemodialysis centers; 3 of them dependent on the Andalusian Health System and the remaining 4 centers belonging to the company Fresenius Medical Care. For each patient, demographic, clinical and monthly monitored biochemical characteristics were considered as well as CKD-MBD treatment. Only laboratory tests containing PTH (measured with the same assay), P and, Ca concentrations, were included in the analysis. In addition to the serum concentration of Ca, P, PTH and alkaline phosphatase, the following set of variables were selected: age, gender, dialysis vintage. Time was entered as another variable together with the corresponding biochemical values and treatment of that specific date. The type of treatment based on the administration of nutritional Vitamin D, Calcitriol, Paricalcitol, Calcimimetics, Calcium and non-calcium based phosphate binders was done following the demands of each patient. In general we followed the KDIGO guidelines for CKD-MBD [2]. Related drugs treatment were associated to 8 binary variables representing whether a patient received a specific drug type during the 30 days before the blood sample for monthly lab was taken.

Initially data contains 46,141 measures of biochemical parameters. Only patients with complete information (Ca, P, PTH, age, gender, dialysis vintage, and type of treatment) were included in the analysis (S1 Dataset).

The study was approved by the Ethics Committee Institutional Review (Comité Ético de Investigación del Hospital Universitario Reina Sofía). Written informed consent was given by patients to be included in the Nephrology Department Data Base; all records / information was anonimyzed and de-identified prior to analysis.

### Random Forest for Data Analysis

**Fig 1:**
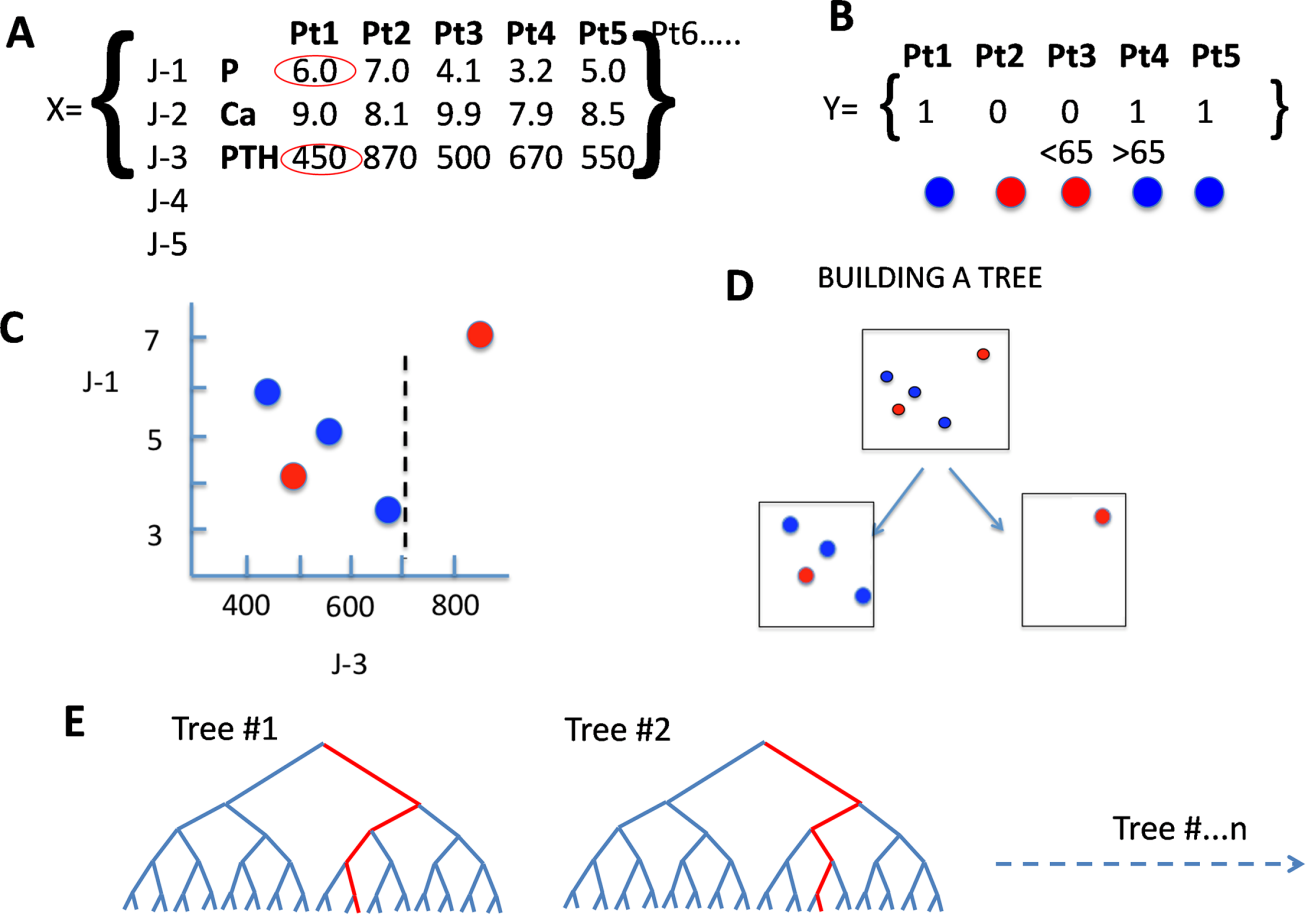
Brief Description Random Forest. (**A)** Dataset. (**B)** Outcome variable; (**C and D)** generation of decision trees. (**E)** Decision Trees. (A) The dataset “X” includes all variables (J-1, J-2….J-n) such as serum levels of P, Ca, PTH, alkaline phosphatase, etc. The values of these variables are obtained from all patients included in the study: Pt1, Pt2, Pt3 up to Ptn. A first step is to construct “intelligent” decision trees based on the available data. (B) Let´s construct a decision tree that predicts an output variable Y that in this case will be age. Open and closed circles represent patients with age < 65 and >65 respectively). Decision trees will be constructed based on the values of many variables from many patients. (C) A set of two variables, J1 (P) and J3 (PTH) are chosen at random from a subset of data that is also chosen at random out of the entire dataset. The values of P are plotted against the corresponding PTH; open and closed circles represent patients with age < 65 and >65 respectively. The best PTH value that discriminate reds from blues is 700. (D) The first discrimination of the decision tree is that with a PTH greater than 700 all (100%) of patients are less than 65 and 75% of patients with less that 700 are more than 65. The next step will be to separate each one of the two groups according to any other variable chosen at random that could be serum calcium or any other. (E) This process will be done using, large number patients and will be repeated many times in different subsets of data at random. A large number of decision trees are constructed; as many as needed to obtain reliable predictions. Further information may be obtained in Antonio Criminisi, Jamie Shotton, Duncan Robertson, and Ender Konukoglu (Anatomy Regression Forests for Efficient Detection and Localization in CT Studies B. Menze et al. (Eds.): MCV MICCAI 2010 Workshop, LNCS 6533, pp. 106–117, 2011.c_Springer-Verlag Berlin Heidelberg 2011).

The information obtained through the Random Forest (RF) analysis is based on a process of learning. A brief description of the RF procedure will uncover the advantages of this data analysis (Fig 1). Decision trees are constructed based on the values of many variables from many patients. Having enough data allows the construction of a large number of decision trees; as many as needed to obtain reliable predictions. In the present study the number of 100 trees was selected for generation of models. The output of RF is the average obtained from all different trees.

RF regression models for the prediction of Ca, PTH and P serum levels were derived.

In the model for prediction of Ca (the output or dependent variable), the rest of variables were used as input variables. The same procedure was applied to obtain models for the prediction of PTH and P. The RF model associates to every possible combination of input variables (e.g., PTH, phosphate, age, use of Calcimimetics, etc.) the prediction of a value for one output variable (e.g., calcium level). Seventy percent of the data is randomly selected to obtain models (training set), the remaining 30% is used as testing set to evaluate the prediction accuracy on un-seen examples (testing set). Accuracy is measured as the mean absolute error and the Pearson’s correlation coefficient calculated considering the estimated values (model output) and the real values of Ca, PTH and P serum concentration. If the model is able to accurately predict the output variable, it can be used to predict the effect of changing the values of input parameters (e.g., taking or not a drug) on the output variable. It is possible to calculate the relative relevance of each input variable in the prediction of a model: this is called feature importance analysis.

The analyses were performed using Matlab version 8.2 (R2013b). In particular TreeBagger algorithm of the Statistics Toolbox was used for RF computation and Regress algorithm was used for linear regressions computation and for Pearson’s correlation coefficient.

## Results

RF models for the prediction of PTH, Ca, and P were computed and optimized. Table 1 shows the characteristics of the study population at the baseline (first available measurement of biochemical parameters).

**Table 1:** Baseline characteristics of patients included in the study.

**DEMOGRAPHIC VARIABLES**	
Male (%)	59.2
Age. Mean (SD)	59.8 (16.6)
Vintage, days. Mean (SD)	3300 (2560)
Fistula (%)	75.5
**BIOCHEMICAL PARAMETERS. MEAN (SD)**	
Calcium (mg/dL)	9.4 (0.9)
Phosphate (mg/dL)	4.7 (1.4)
PTH (pg/mL)	317 (320)
Alkaline Phosphatase (IU/L)	142 (126)
Potassium (mmol/L)	5.3 (0.9)
**CKD-MBD RELATED DRUGS ADMINISTRATION(% of patients on the medication)**	
D-Vitamin (%)	1.4%
Calcitriol (%)	15.6%
Paricalcitol (%)	13.1%
Calcium Binder (%)	42.4%
Magnesium Binder (%)	0.6%
Sevelamer Binder (%)	41.8%
Lantano Binder (%)	10.6%
Calcimimetics (%)	15.5%

CKD-MBD: Chronic kidney disease-Metabolic bone disease; SD: Standard deviation

The values of output variables calculated from models obtained by RF were correlated with actual values obtained from the database. The correlation coefficients between the predicted output values and the real values were the following: Calcium model: R_Ca_ = 0.60 (p<0.001); PTH model, R_PTH_ = 0.67 (p<0.001); phosphate model, R_P_ = 0.51 (p<0.001). These correlation coefficients are superior to those obtained using models derived from multivariate linear analysis: R_Ca_ = 0.37 (p<0.001), R_PTH_ = 0.47 (p<0.001) and R_P_ = 0.37 (p<0.001).

**Fig 2:**
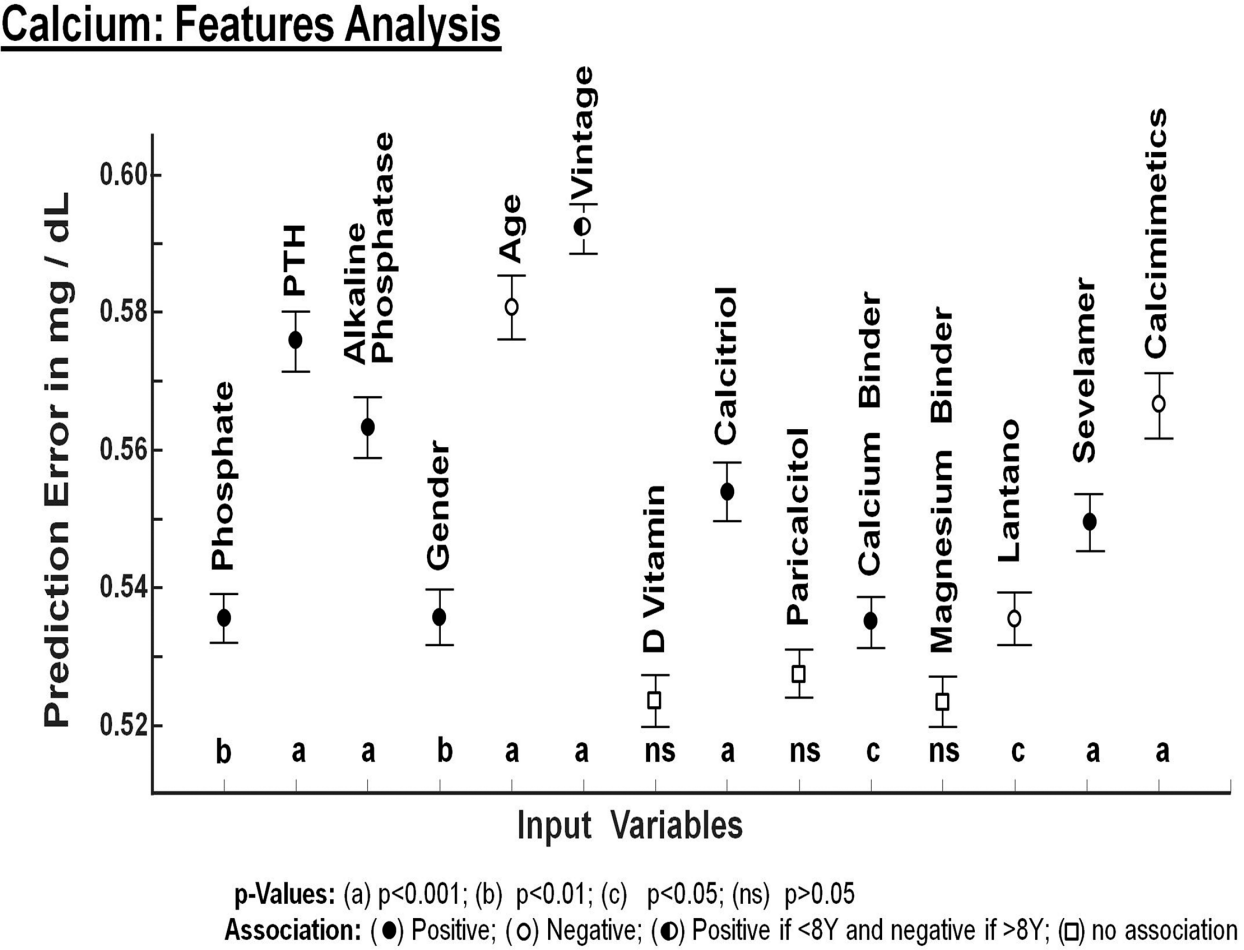
Prediction error for Serum Calcium concentration by each one of the variables included in the analysis. The vertical axis of Fig 2 depicts the error in prediction of serum calcium concentration (mg/dl) that will be caused if specific input variable is removed from the mathematical model generated by Random Forest. The magnitude of prediction error obtained with each variable is being compared with the rest of the variables. Only variables that show a significant effect in predicting values of calcium were included in this analysis.

**Fig 3:**
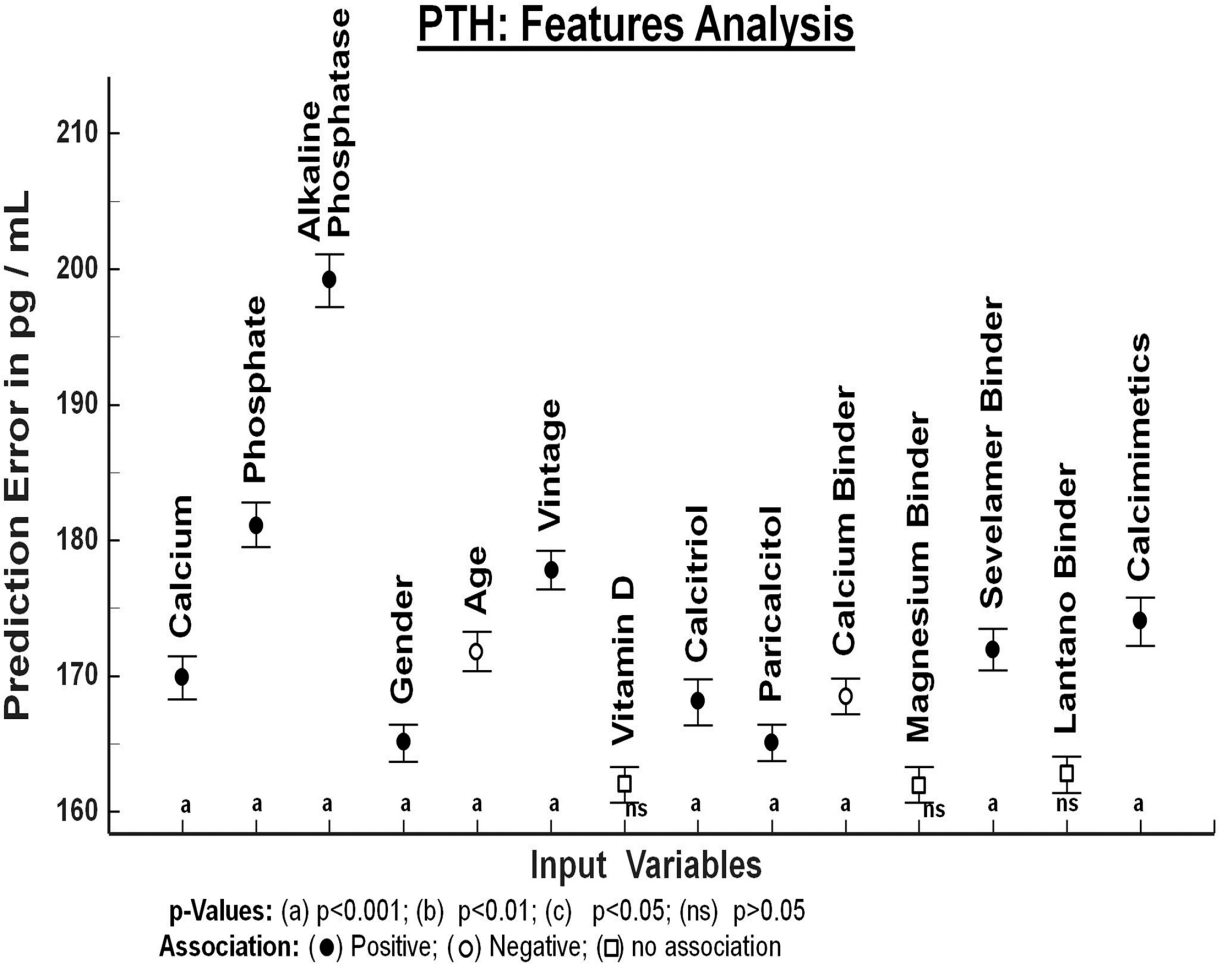
Prediction error for Serum PTH concentration by each one of the variables included in the analysis. The vertical axis of Fig 3 depicts the error in prediction of serum PTH concentration (pg/ml) that will be caused if specific input variable is removed from the mathematical model generated by Random Forest. Paricalcitol, Calcitriol and Calcimimetics are positively associated with PTH because this therapy is given to patients with hyperparathyroidism. The magnitude of prediction error obtained with each variable is being compared with the rest of the variables. Only variables that show a significant effect in predicting values of PTH were included in this analysis.

**Fig 4:**
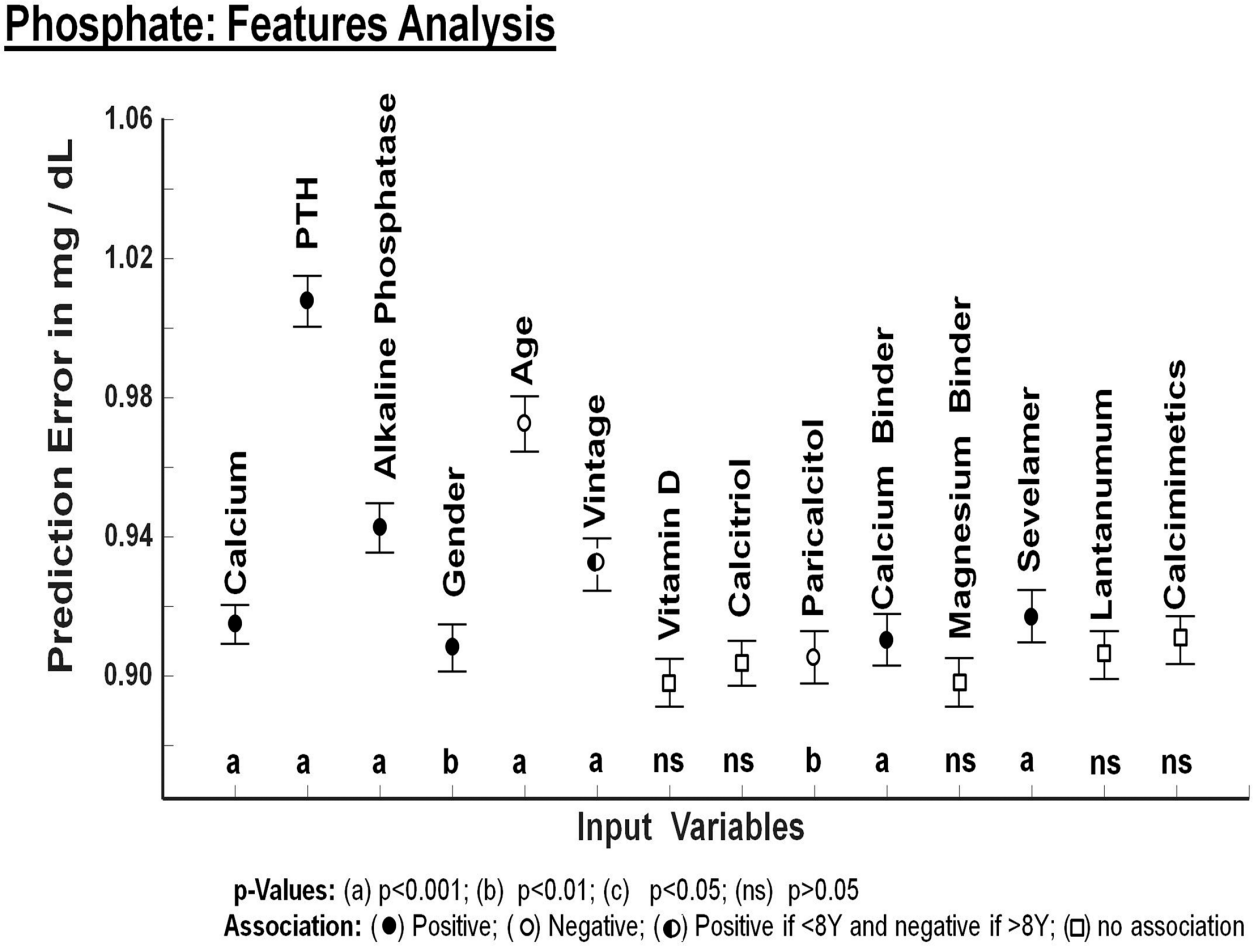
Prediction error for Serum Phosphate concentration by each one of the variables included in the analysis. The vertical axis of Fig 4 depicts the error in prediction of serum phosphate concentration (mg/dl) that will be caused if specific input variable is removed from the mathematical model generated by Random Forest. Sevelamer and calcium binders are positively associated with serum phosphate concentration because patients with hyperphosphatemia receive phosphate binders. The magnitude of prediction error obtained with each variable is being compared with the rest of the variables. Only variables that show a significant effect in predicting values of phosphate were included in this analysis.

Then, the RF models were explored through a feature importance analysis which evaluates the role of each input variable on the prediction of the output variables: Ca, PTH and P (Figs 2, 3 and 4). The relevance of each feature variable is assessed by dropping the variable from the model and evaluating to what extent the prediction error increases. If a variable is relevant, the model without that input variable will have a substantial increase in prediction error. The degree of increase on prediction error is a measure of the relevance of that input variable in the model. (Figs 2, 3 and 4).

### Calcium model

Analysis of variable importance in the prediction of serum calcium concentration revealed that the most relevant variables are the serum concentrations of PTH, alkaline phosphatase, dialysis vintage and age (Fig 2). The vertical axis of Fig 2 depicts the ±error value in the prediction of serum calcium concentration (mg/dl) that will be caused if specific input variable is removed from the mathematical model generated by RF. Of interest is the fact that, removing age and vintage from the model will cause an error in prediction of serum calcium of ±0.57 and ±0.58 mg/dl respectively. Serum Ca level is also closely and positively associated with serum concentration of PTH and alkaline phosphatase. The association between serum Ca and P levels is very modest.

Calcimimetics and calcitriol appear to be associated to low and high calcium levels respectively. The administration of calcimimetics was associated to a decrease in serum calcium to a mean value of 9.0 mg/dl. Calcitriol increased the mean serum calcium to 9.8 mg/dl. The association between Paricalcitol and serum calcium level was modest. (Fig 2). None of the phosphate binders, except sevelamer, was associated to the value of serum calcium. Patients on sevelamer show serum calcium levels slightly higher than patients not taking sevelamer. This may reflect the fact that sevelamer was given to patients with high calcium to avoid calcium containing calcium binders.

The comparison of prediction error among variables included in the feature analysis for calcium is shown in Table 2.

**Table 2:** Comparison of prediction error among variables included in the feature analysis for calcium.

	PTH	AP	G	Age	Vint.	CTR	Ca Bind.	Sev	CM
**P**	<0.001	<0.001	ns	<0.001	<0.001	<0.001	ns	<0.001	<0.001
**PTH**	-	<0.001	<0.001	ns	<0.001	<0.001	<0.001	<0.001	<0.001
**AP**	-	-	<0.001	<0.001	<0.001	<0.001	<0.001	<0.001	ns
**G**	-	-	-	<0.001	<0.001	<0.001	<0.001	<0.001	<0.001
**Age**	-	-	-	-	<0.001	<0.001	<0.001	<0.001	<0.001
**Vint**	-	-	-	-	-	<0.001	<0.001	<0.001	<0.001
**CTR**	-	-	-	-	-	-	<0.001	Ns	<0.001
**Ca Bind.**	-	-	-	-	-	-	-	<0.001	<0.001
**Sev.**	-	-	-	-	-	-	-	-	<0.001

Abreviations: (P) Phosphate; (AP) Alkaline Phosphatase, (G) Gender, (Vint) Vintage, (CTR) Calcitriol, (Ca Bind.) Calcium binders, (Sev.) Sevelamer. (CM) Calcimimetics

### PTH model

Variables that were associated with serum PTH concentration are shown in Fig 3 The vertical axis depicts the error in prediction of serum PTH concentration (pg/ml) that will be caused if specific input variable (horizontal axis) is removed from the mathematical model generated by RF. As expected, values of serum PTH concentration were closely associated to alkaline phosphatase. PTH was positively associated with dialysis vintage and negatively associated with age. There was also a noticeable positive association between serum concentrations of PTH and P that is clearly superior to the association PTH-Ca. In patients receiving calcimimetics the average serum PTH concentration was 423 pg/ml. PTH was also associated to the use of Calcitriol and Paricalcitol; patients already on calcitriol had mean PTH values of 434 pg/ml while those on paricalcitol had a mean PTH of 359 pg/ml.

The comparison of prediction error among variables included in the feature analysis for PTH is shown in Table 3.

**Table 3:** Comparison of prediction error among variables included in the feature analysis for PTH.

	P	AP	G	Age	Vint.	CTR	Paric	Ca Bind.	Sev	CM
**Ca**	<0.001	<0.001	<0.001	ns	<0.001	ns	<0.001	ns	ns	<0.001
**P**	-	<0.001	<0.001	<0.001	<0.001	<0.001	<0.001	<0.001	<0.001	<0.001
**AP**	-	-	<0.001	<0.001	<0.001	<0.001	<0.001	<0.001	<0.001	<0.001
**G**	-	-	-	<0.001	<0.001	0.016	ns	ns	<0.001	<0.001
**Age**	-	-	-	-	<0.001	<0.001	<0.001	<0.001	ns	ns
**Vint**	-	-	-	-	-	<0.001	<0.001	<0.001	<0.001	<0.001
**CTR**	-	-	-	-	-	-	<0.001	ns	0.001	<0.001
**Paric**	-	-	-	-	-	-	-	0.001	<0.001	<0.001
**Ca Bind**	-	-	-	-	-	-	-	-	<0.001	<0.001
**Sev**	-	-	-	-	-	-	-	-	-	ns

Abreviations: (P) Phosphate; (AP) Alkaline Phosphatase, (G) Gender, (Vint) Vintage, (CTR) Calcitriol, (Paric) Paricalcitol, (Ca Bind.) Calcium binders, (Sev.) Sevelamer. (CM) Calcimimetics, (Ca) Calcium

#### Predicting the change in PTH resulting from a variation of serum Phosphate

**Fig 5:**
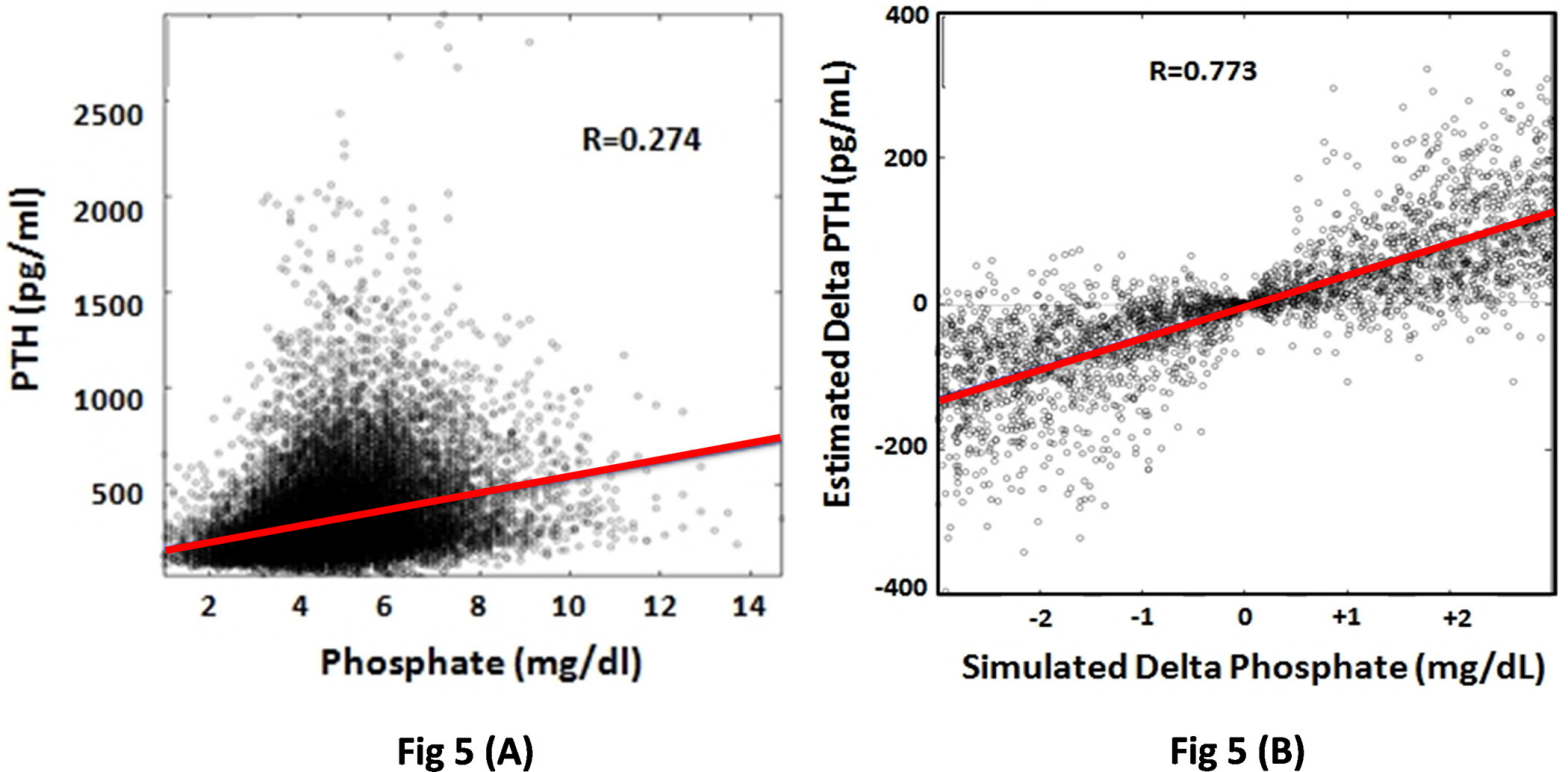
(A) Correlation of Serum PTH and phosphate concentration. (B)Estimated change in PTH concentration in response to a simulated change in serum phosphate concentration. These estimations are derived from Random Forest regression models for the prediction of outcome variables.

The positive association between serum PTH concentration and serum phosphate levels was further analyzed using two different approaches. First, classical linear regression analysis shows that the serum concentrations of PTH and P are significantly correlated: R = 0.27, p<0.001 (Fig 5A). Second, using the mathematical models derived from RF data analysis we predicted the change in PTH resulting from a variation in serum P concentration. A random variation was imposed to P (from -3 mg/dl to +3 mg/dl) and the difference between estimated PTH before and after P variation was computed (Fig 5B). This approach reveals a strong relationship between the changes in serum P and PTH: R = 0.77 (p<0.001). According to these analyses a decrease in serum P of 2 mg/dl may be associated to a change in PTH of +50 to -250 pg/ml, as shown in Fig 5(B).

### Phosphate model

Variables that were associated with serum P concentration are shown in Fig 4. Serum phosphate concentration was positively associated with serum PTH and negatively associated with age. Serum P level was associated to the use of phosphate binders: calcium binders, sevelamer and lanthanum without significant differences among the phosphate binders

The comparison of prediction error among variables included in the feature analysis for phosphate is shown in Table 4.

**Table 4:** Comparison of prediction error among variables included in the feature analysis for phosphate.

	PTH	AP	G	Age	Vint.	Paric	Ca Bind.	Sev
**Ca**	<0.001	<0.001	ns	<0.001	<0.001	ns	Ns	ns
**PTH**	-	<0.001	<0.001	<0.001	<0.001	<0.001	<0.001	<0.001
**AP**	-	-	<0.001	<0.001	ns	<0.001	<0.001	<0.001
**G**	-	-	-	<0.0001	<0.001	ns	Ns	ns
**Age**	-	-	-	-	<0.001	<0.001	<0.001	<0.001
**Vint.**	-	-	-	-	-	<0.001	<0.001	<0.001
**Paric**	-	-	-	-	-	-	Ns	ns
**Ca Bind.**	-	-	-	-	-	-	-	ns

Abreviations: (PTH) Parathyroid Hormone (P) Phosphate; (AP) Alkaline Phosphatase, (G) Gender, (Vint) Vintage, (CTR) Calcitriol, (Paric) Paricalcitol, (Ca Bind.) Calcium binders, (Sev.) Sevelamer. (CM) Calcimimetics, (Ca) Calcium

## Discussion

The aim of the present study is to investigate the utility of the new data analysis system Random Forest to quantify associations between parameters of mineral metabolism in HD patients. The parameters evaluated were the serum concentrations of Ca, P and PTH, parameters that guide the physician in deciding therapy in daily practice [1,2,21,22,23,24,25]. One of the basis of the present study is the assumption that these three parameters should not be evaluated individually because the processes involved in their regulation are common, thus changes in one parameter will affect the others. One should be open to accept that mathematical models, other than classical linear statistical analysis, may be helpful to analyze variables that are closely related to each other.

While mutual influences between serum levels of Ca, P and PTH are expected, it is difficult to quantify to what extent these parameters may influence each other because of the interference of confounding factors. RF analysis can identify non-linear relationships among many variables without any a priori assumption, i.e. the probability distribution that characterizes the phenomenon is automatically estimated by the data itself, while with a classical analytical approach the knowledge of data distribution has to be assumed *a priori* [16,17]. Therefore, taking into account the complexity and non-linearity involved in the CKD-MBD scenario the use of an advanced RF model resulted in a significant improvement in extracting useful information from the available data. In particular through the derived predictive models of Ca, PTH and P it was possible to identify interesting patterns of associations among different parameters and therapies. Even if a specific association is not straightforward, the predictive model is able to mediate the concurrent effect of all involved features making the association to emerge clearly. Predictive models may eventually help clinical diagnosis and eventually therapeutic decisions. Evidently final decisions must include bedside assessment of patient condition and we are far away from obtaining valid algorithms that could replace an experienced Doctor. Further studies will be necessary to prove that these advanced methods of analysis have substantial value in assessing complex relationships of various parameters in a single patient. If that is the case, this type of information may help to decide therapeutic interventions. The Ca, PTH and P models obtained by RF do not expose any previously unknown association, however these models allows to quantify to what extent one independent variable is needed to optimally predict values of the outcome variable.

The study reveals that serum calcium is positively associated with time on dialysis but the association becomes negative after 8 years on dialysis. This finding is explained by the fact that serum calcium is negatively associated with age; it is likely that after several years of dialysis, patients are older and the serum calcium decreases. Therefore this finding is explained by the negative age effect overcome the positive effect of time in hemodialysis. What is really relevant is that removing age and vintage from the model that predict serum Ca will cause an error of 0.57 and 0.58 mg/dl respectively (Fig 2 and Table 2). The Serum Ca concentration is directly related to both PTH and alkaline phosphatase suggesting that in most patients PTH drives the serum Ca concentration. Without PTH or alkaline phosphatase the error in predicting Ca would be more than 0.55 mg/dl. Ca is also associated to the use of calcimimetics and vitamin D analogs. [26,27,28].

This study found that PTH was positively associated with dialysis vintage (Fig 3 and Table 3); this is not an unusual finding since there are reports showing that hyperparathyroidism progresses with time on dialysis until the patient becomes older. Age, is known to be negatively associated with PTH and with a tendency to adynamic bone disease [10,29].

The close association between PTH and alkaline phosphatase illustrates the consistency of the RF technique used in the present work. It was interesting to find out that the PTH-P association was superior to the PTH-Ca association.

The positive association between serum PTH concentration and serum P levels is shown in Fig 5(A) and 5(B). Plotting the absolute values of serum PTH and P concentration (Fig 5A) shows a correlation that although significant is not valid to predict changes in PTH concentration based on changes in serum P level. The prediction of a change in PTH resulting from a modification of serum P is show in Fig 5(B), this correlation is obtained using RF approach. This figure shows the correlation between PTH and P being analyzed as the deviation of PTH keeping fixed all the features and varying just P. The results of this analysis reveal a strong correlation between the modifications in PTH and P values. A modification in one of these two parameters should be accompanied by a corresponding variation in the other parameter [2,18,30]. High serum P could be associated to high PTH through different mechanisms: a high P level may directly increase PTH secretion and synthesis, high phosphate also produces skeletal resistance to PTH [31,32]. Additionally high PTH level increases bone efflux of P that contributes to hyperphosphatemia. This strong association between PTH and P suggest that strategies to decrease PTH and P should be combined.

High PTH levels were associated to the use of calcimimetics, this association is expected since calcimimetics are used to reduce PTH levels [26,27]. Calcitriol and paricalcitol were also associated to values of PTH, however it was not as strong as the PTH-calcimimetics association.

Values of serum P concentration were negatively associated with age. This has been reported previously [33] and it has been attributed to a decrease in protein intake. Interestingly, it appears, from the present analysis, that age may influence serum concentration of PTH, P and also Ca but guidelines do not mention any type of correction for age. It may be appropriate to investigate whether there is an age level at which these parameters are being significantly modified. The majority of patients were on one or more types of phosphate binders and, according to the performed analyses, it appears that the type of binder is not linked to a given phosphate level.

In conclusion the analysis of complex interactions between the different parameters of mineral metabolism may benefit from more advanced mathematical approach such as Random Forest which is able to identify and computes non-linear relationships among many variables without any *a priori* assumption.

## Supporting Information

S1 Dataset Compilation of data. (ZIP)
