# Supplementary figures and images for: A New Data Analysis System to Quantify Associations between Biochemical Parameters of Chronic Kidney Disease-Mineral Bone Disease
Source: PLoS One. 2016 Jan 25;11(1):e0146801. doi: 10.1371/journal.pone.0146801 (PMC4726537; doi:10.1371/journal.pone.0146801)

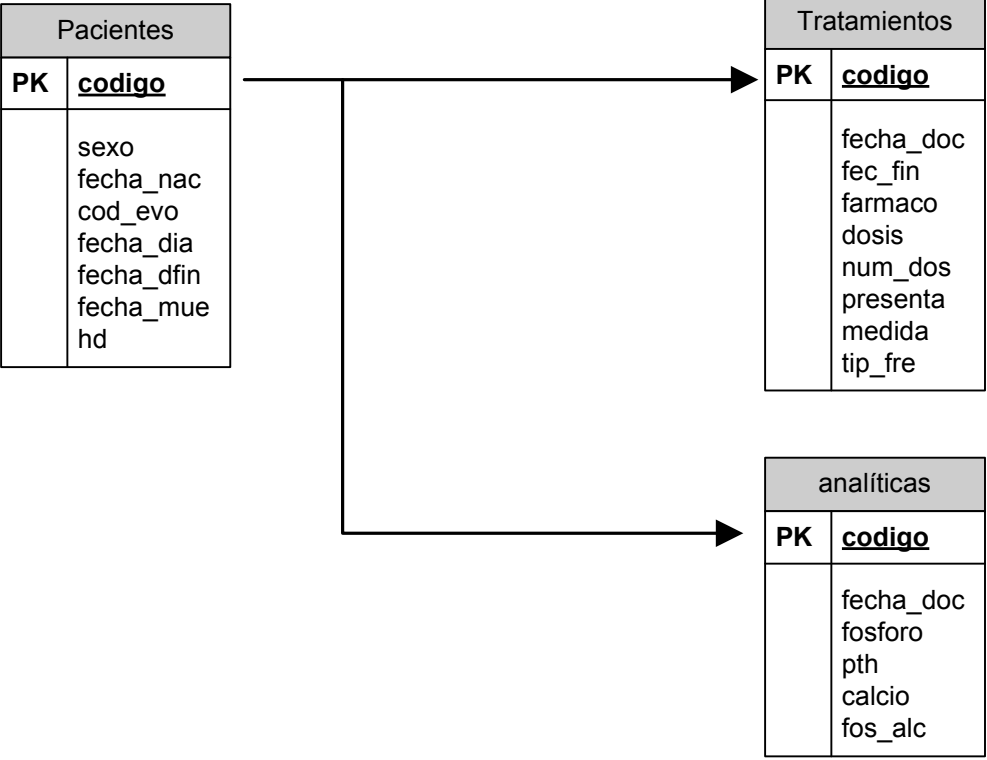

Supplement: S1 Dataset — (ZIP) [file pone.0146801.s001.zip › Visio-Dibujo1.pdf]
